# Determining client need in a multi-state fetal alcohol syndrome consortium: from training to practice

**DOI:** 10.1186/1744-9081-3-10

**Published:** 2007-02-15

**Authors:** Suzanne Christopher, Tim Dunnagan, George Haynes, Lili Stiff

**Affiliations:** 1 Department of Health and Human Development, Herrick Hall, Montana State University, Bozeman, MT 59717, USA; 2 Department of Health and Human Development, Haley Road, Bozeman, MT 59715, USA

## Abstract

**Background:**

A multi-state consortium was developed in the US to conduct baseline data collection and intervention research on fetal alcohol syndrome. Each state employed support specialists whose job it was to reduce or eliminate alcohol consumption in women who were at high risk for drinking alcohol during their pregnancy. The purpose of this paper is to report how support specialists in three primarily rural/frontier states were trained to assess client need and how client need was actually assessed in the field.

**Methods:**

A qualitative process evaluation was conducted using semi-structured interviews. Interviews were conducted with state staff involved in support specialist training and consortium activities and the support specialists themselves. Inductive analyses were conducted with interview data.

**Results:**

Need determination varied by state and for one state within the state. How support specialists were trained to assess need and how need was assessed in the field was mostly congruent.

**Conclusion:**

Process evaluation is an effective method for providing practical and useful answers to questions that cannot be answered by outcome evaluation alone.

## Background

Determining or assessing need is seen as "perhaps the most critical part of program planning" [1] (p. 74). A primary goal in determining need is to find the gaps between what currently exists and what could be, what is desired, or what is an established standard [2,3]. Results from a need determination provide a focus for programs and for intervention strategies [4]. If a person is at or above what could be, is desired, or the established standard, there is no need; if they are below, there is need. There are a multitude of methods to determine client and community need.

This paper reports on a process evaluation study that assesses the difference between how paraprofessional support specialists were trained to assess need and how they actually assessed client need in the field. The support specialists were working with a multi-state fetal alcohol syndrome prevention project in the US. This multi-state group is hereafter referred to as the consortium. Both the perspectives of the individuals who developed and implemented the training and the perspectives of the support specialists who worked with high risk pregnant women were taken into account. Differences and similarities among states and between trainers and support specialists regarding determining client need were assessed.

Needs assessment techniques were chosen by state versus collectively across the three states. Representatives from each state met together to discuss potential needs assessment strategies and techniques. After this meeting, staff members from individual states convened with an awareness of many needs assessment options and selected the techniques that best fit the needs of their state. Therefore, each state was free to select the tools and techniques that were most appropriate for their population and settings.

Although there are a number of articles that discuss how to conduct needs assessments around various health topics and articles that discuss the results of needs analyses, we were not able to locate any articles that compare the differences between how service providers are trained to conduct needs assessments and how those assessments are actually carried out in the field. The results of this paper have implications for the multitude of health and human service programs that train providers in methods for conducting individual-level needs assessments.

As in many other programs, needs assessments for this program were used to target specific intervention strategies to meet identified needs. The research presented in this paper provides valuable process evaluation information that can be used to gain a deeper understanding of the activities and results of the intervention strategies. Quantitative outcome evaluation data can help to answer the question "did something change that was statistically significant as a result of the intervention?" For example, did the intervention change pregnant women's drinking behaviors or levels of social support, depression, or family functioning? Qualitative process evaluation data can help program staff understand why changes did or did not occur as shown in the quantitative analyses [5-7]. Using a hypothetical example, qualitative data can help staff understand why social support and depression changed but family functioning and drinking behaviors did not. It is fairly common for intervention strategies aimed at changing multiple outcomes to have varying levels of success across outcomes.

Previous analyses of this program have examined two important process evaluation questions: (1) Did people needing help for alcohol abuse receive more time from the support specialists[8]; and (2) What were the characteristics of pregnant women most at-risk for alcohol consumption[9]. A forthcoming paper will answer the important outcome evaluation question of whether or not the intervention reduced alcohol abuse by high-risk pregnant women.

## Methods

### Question construction

Questions were initially developed with the purpose of gathering need information that was not being garnered through other consortium-based data collection methods. Questions were reviewed by members of the consortium and minor revisions were made based on the review. The questions were open-ended and semi-structured, allowing the respondents to share information without the restrictions of closed-ended or forced choice questions[10]. That they were semi-structured (i.e. the same open-ended questions were asked of each set of respondents) allowed for comparisons among those individuals interviewed[11,12].

### Data collection

Two groups of individuals were interviewed from each of the three states. For reasons of confidentiality, in this paper we will refer to the states as State A, State B, and State C. The first group consisted of individuals who were involved in developing and delivering support specialist training. Anywhere from one to three individuals were interviewed from each state. The second group was the support specialists themselves. There were two support specialists interviewed in State A, four in State B, and two in State C. This includes all of the support specialists except one specialist in State C who was hired immediately prior to data collection. The support specialists are paraprofessionals who have prior work and/or life experience that would enable them to successfully work with high-risk pregnant women in their communities.

Interviews were conducted via telephone in the Fall of 2002 with additional information gathered through follow-up e-mail correspondence and from the support specialist training and field work documents. The use of two different types of qualitative data, interviews and documents, strengthens this study by providing data from multiple sources[13].

To gather information on support specialist training and practice in States A and C, telephone conversations and e-mail correspondence was conducted between the first author and staff. The first author and staff developed and conducted training and were involved in providing needs assessment support to the support specialists in those states. The first author was deeply involved in developing and conducting the support specialist training in State B. Most of the training information for State B came from her experience. In addition, State B hired outside consultants to help with the training. The primary consultant and state staff provided additional information.

Interviews with support specialists in all three states were conducted by a graduate student at Montana State University who was completing a Master's degree in counseling and had extensive open-ended interviewing experience. She received additional interviewing training from the first author.

The trainers/developers were asked how they trained their support specialists to determine client need. Follow-up probes were used to elicit detailed information on the training. For example, they were asked how the training was carried out, if role playing was used, and the duration of the training process. Next, they were asked what written material in the training curriculum pertained to determining client need. Finally, they were asked about how need determination happens in the field. Documents were obtained whenever possible to provide additional information related to the questions.

Support specialists were asked to describe the training and/or information they were given to help them decide what the needs were of the women they work with. They were specifically asked this question in the context of the training/workshops they received to help them prepare for their job. Probes were used to gather additional information. Probes included asking about written materials they received and how the training was carried out (e.g., role play, lecture). Examples of need were given to clarify the type of information desired. Next, support specialists were asked how they determined need when they were working directly with women. Probes were used to clarify if this was done alone or with others and when this happened in the process of working with clients. Detailed examples of determining need were asked for.

Finally, support specialists were asked about need determination differences between what they received from training compared with what happens in the field. Questions were asked to determine how they felt that their training was helpful and useful and to ascertain additional methods of need determination other than those provided for in their training.

### Data analysis

Hand written or typed notes were taken during each telephone interview. Hand-written notes were later typed into a computer using word processing software. Notes were expanded on and made more complete immediately after the interview. The "period after an interview or observation is a critical time of reflection and elaboration"[13]. Existing documents were reviewed for relevant content.

Inductive analysis was conducted based on methods described by Strauss and Corbin[14,15], Patton[11,12], and Bogdan and Bicklen[16]. In inductive analysis, themes arise from the data versus being predetermined before analyses begin[17]. The transcript was read, themes and specific answers to questions were extracted and example quotes were identified. To increase the validity of the analysis, member checking was performed. In member checking, data interpretation and results are tested against the perception of the respondents[18]. "Validity in qualitative research has to do with description and explanation, and whether or not a given explanation fits a given description. In other words, is the explanation credible?"[19] (p. 216). Results and interpretation of data were sent to interviewees for verification. Changes in text were made when necessitated.

## Results

Results are presented by state with information on determining client need from the training displayed first. Information on determining client need in the field follows. For each state, results from state staff are provided first followed by information from the support specialists. Table 1 presents a comparison of the differences and similarities between needs assessment activities conducted by the three states in formal training versus in the field.

**Table 1:** Comparison of needs assessment activities conducted by states in formal training "train" and in the field "field."

**Needs Assessment Activity**	**Other educational resources**		**Difference Game**		**Assessment instruments**		**Discussion between ss and clients**		**Support, care, or personal plan**		**Discussion between ss and other staff**		**Motivational Interviewing**	
	Train	Field	Train	Field	Train	Field	Train	Field	Train	Field	Train	Field	Train	Field

*State A*														
Staff	No	No	No	No	**Yes**	**Yes**	No	**Yes**	**Yes**	**Yes**	No	**Yes**	**Yes**	**Yes**
Support Specialist (SS)	No	No	No	No	**Yes**	**Yes**	No	No	No	**Yes**	No	No	No	**Yes**
*State B*														
Staff	No	No	**Yes**	**Yes**	No	**Yes**	No	No	No	**Yes**	No	No	**Yes**	No
Support Specialist	No	No	**Yes**	**Yes**	No	No	No	**Yes**	No	No	No	No	**Yes**	**Yes**
*State C*														
Staff	No	No	No	No	**Yes**	**Yes**	**Yes**	**Yes**	No	**Yes**	No	**Yes**	**Yes**	No
Support Specialist	**Yes**	**Yes**	No	No	**Yes**	**Yes**	No	**Yes**	**Yes**	No	No	**Yes**	**Yes**	No

Other educational resources: educational publications and information from other agencies.

Difference Game: an interactive game that enabled clients and social workers to identify and prioritize client needs.

Assessment instruments: consortium evaluation tools such as the Addictions Severity Index.

Discussion between ss and clients: informal discussions outside other needs assessment activities.

Support, care or personal plan: a document that outlined intervention activities for the client in the FAS intervention.

Discussion between ss and other staff: conversations between the support specialist and state site staff, supervisors or trainers.

Motivational interviewing: specific interviewing techniques used to assess need.

### Determination of client need in training

#### State A staff

Training for determining client need in State A focused on reviewing the assessment instruments and how to collect data from the instruments and on reviewing the process whereby support specialists would receive a support plan outlining client need from state staff. Supplementary need assessment information came from training in case management, motivational interviewing, and domestic violence. In the training, the trainer reviewed the assessment instruments and how data was to be collected. All but one of the data collection instruments were completed by the client. Support specialists were present during this time to provide clarification. The Addictions Severity Index (ASI) was completed via interview between the support specialists and the clients. Therefore, training on the ASI was more extensive than the other instruments. Support specialists were trained on conducting ASI interviews by a trainer who had experience administering the ASI. Support specialists practiced role playing ASI interviews. Case management and motivational interviewing were thought to assist needs assessment by providing the support specialists with skills for engaging clients, providing lists of resources in the community, and providing training on when to refer clients to those resources.

#### State A support specialists

Both support specialists discussed that the training for need determination focused on the administration of the assessments, primarily the ASI. Time was spent in the training on test administration versus a specific training in assessing certain types of need (e.g. assessing the need for housing assistance). The support specialists remembered spending part of the training practicing and role playing completing the assessments. Specialists remarked that the training format included practicing the test administration in a participatory fashion and having question and answer sessions about the assessments. Written materials received in the training consisted of copies of the assessments and the core curriculum.

### Determination of client need in the field

#### State A staff

When clients entered the program they completed a battery of data collection instruments. Assessments were also completed once during the 1^st^, 2^nd^, and 3^rd ^trimester and approximately 3 months after the infants were born. Support specialists were in charge of data gathering for the intervention group. Information was gathered on social support, family functioning, substance use and abuse, depression, self-efficacy, and general health status. Not all assessments were done at each data gathering time period.

Support specialists mailed the completed assessments back to state staff. A staff member, who is a licensed master's prepared social worker reviewed the assessments. Based on information from all of the assessments and a conference with a psychiatrist, the staff member put together a support plan highlighting areas of client need. This support plan was sent back to the support specialists within 2–3 days of receipt of the assessments. The support plan included scores on all of the assessment instruments over time and specific areas of client need derived from scores on the assessments. Support specialists shared the support plans with clients who signed off on the plan.

The wording used in the support plan mirrored language suggested by motivational interviewing proponents exhorting support specialists to explore, encourage, and assist clients. Again, this language went along with the fact that the support specialists were not trained therapists; they were in the client's home to provide support and encouragement, not therapy. State staff prepared a set of responses to specific issues raised in the assessments to add consistency to the support plans. Additionally, specific common activities were recommended for the support specialist to implement with clients. The support plan was updated each time the client completed an assessment battery. Additionally, because of the training in motivational interviewing, state staff believed that assessments happen informally throughout the relationship between support specialists and clients.

In addition to the support plan, state staff were in direct contact with support specialists on a frequent basis to discuss client progress, client need, and adherence to study protocol. The state staff did not see the support specialists as the main determiners of client need; they saw the assessments and the support provided to them by state staff as the main determiners of need. Staff emphasized that their support specialists were not trained therapists and were not trained at doing assessments and so were not in a position to determine client need without the assistance of more highly trained personnel.

#### State A support specialists

On the same day assessments were completed, data were entered into computers by the support specialists. By doing this they were "able to see changes in needs, progress and regress in the various areas first hand, and adjust our interviewing and support activities accordingly". This information was then sent to the state staff. Need was assessed by the state staff who generated a support plan and sent the plan back to the support specialist. This plan served as a guideline for addressing need, but specialists felt that they could deviate from this plan. One specialist said that she showed the plan to her clients and asked, "Does this fit you? Are there things we have missed?" The support plan was supplemented as new needs arose in weekly interactions. One specialist stated that the participant reviewed the support plan with her and that they could modify or amend the needs assessment portion. Specialists felt this was flexible and used a common sense assessment of need in addition to the support plan. One specialist mentioned that they reviewed and revised the support plan on a weekly basis with the client. This was a scheduled part of each visit.

One of the support specialists stated that learning to assess need happens on the job, learning as the intervention progresses. An example given by a support specialist was a woman whose test results from the ASI showed high levels of substance use. One support specialist mentioned that the ASI is the most useful tool for assessing needs, stating that "women will tell you things on the test they would never tell you in another way." The support specialist felt that in one case the ASI results helped the client to acknowledge the severity of her substance use and the support plan allowed for the treatment program.

One support specialist also mentioned how learning to do motivational interviewing was helpful in allowing clients to refine and redirect parts of the support plan. She also mentioned that motivational interviewing techniques facilitated relationship building, which allowed for a discussion of client need.

### Determining need in training

#### State B staff

In State B, the main method for determining need discussed in the training was the use of the Difference Game, a "card-sort assessment method designed to enable clients and social workers to work together to identify client needs"[20] (p. 429). Some reasons why the staff in State B decided to use the Difference Game was that it was developed to be used with alcohol- and drug-dependent women, it is an interactive, hands-on activity that was thought to engage women in the needs assessment process and it is client-driven versus staff-driven. The Difference Game consists of a set of cards with the statement "It would make a difference in my life if I had..." written on the top of each card. Below are written possible needs that clients may have. There are wild cards which are blank cards that clients may use to list needs not on any of the other cards. The clients sort through the cards putting all of the cards into a yes and no pile. The yes pile consists of things that would make a difference and the no pile things that would not make a difference in their lives. The client then picks and ranks their top five needs.

In the training, the Difference Game and its use were discussed. Support specialists role-played both the part of the client and the part of the support specialist. The written curriculum included a description of how to use the Difference Game and each support specialist was given a set of Difference Game cards to take with them.

Motivational interviewing was also part of the training in State B. It was felt that this would help with the needs assessment process by training the support specialists to help clients elaborate on and discuss needs identified during the Difference Game. There was a written section on motivational interviewing that described principles of motivational interviewing, components of motivational interviewing, strategies to promote change, getting people to talk about change, signs of readiness for change, possible key questions to ask clients, and roadblocks to motivational interviewing.

#### State B support specialists

Support specialists mentioned receiving training in several areas to assist in determining need. The Difference Game and motivational interviewing were mentioned by all of the support specialists. One support specialist stated that the training for motivational "interviewing allowed the support specialist to be able to really hear the needs of the participant." In addition, two of the support specialists discussed role plays that occurred in the training as helping them with needs assessments.

The written material that they remember receiving was around the Difference Game and motivational interviewing. One support specialist stated that they did not receive written material about resources in their area to help with needs, however two support specialists commented about receiving resource lists for their geographic area. The remaining support specialist did not mention resource lists as related to determining need.

### Determining need in the field

#### State B staff

In most sites, the Difference Game was completed on the first or second visit the support specialists had with their clients. In some sites, support specialists were with their supervisors when the Difference Game was completed. In other sites, the support specialists were alone. Most visits occurred in the client's homes. After completing the Difference Game, support specialists filled out a care plan that listed the top five areas of need identified by their clients. Support specialists used this care plan to keep track of client needs, plan for future home visits, and track change in need over time. There was space on the care plan where specialists could note progress for each identified need.

Assessment instruments, including the ASI, were completed by data coordinators. The data coordinators sent the completed instruments to an evaluation team located off-site from the state staff. The support specialists were to receive information on the assessment results from the data coordinators or their supervisors.

#### State B support specialists

All of the support specialists mentioned that needs assessment was something that was on-going and occurred during the entire time that that specialist met with the client. They also mentioned that the process was strongly client driven. The support specialists developed plans for meeting need, but were open to hearing about new needs and encouraged clients to discuss needs that were in addition to those raised by the Difference Game. Three support specialists discussed the use of the Difference Game and stated that it was an excellent method for defining needs. One support specialist said that with the card sort "they really come and start telling you things".

All State B support specialists also discussed that they learned a lot about assessing needs by being on the job and being with their clients. One support specialist mentioned that needs assessment was not her primary goal, that "my primary goal is to develop a relationship and out of the relationship they talk...part of the process is assessing needs." There were many allusions made regarding motivational interviewing. For example, one specialist stated that "I learned how to speak to them and sit there, listen to their needs instead of just jumping in and telling them what to do." All of the support specialists discussed consulting with their supervisors and support staff to help them work more effectively with clients. One support specialist added that a lot of times client need is visibly apparent. She mentioned hygiene, family functioning and a safe environment as areas that were visibly assessed.

### Determining need in training

#### State C staff

Staff in State C stated two primary methods for assessing need. The first occurred before the training when the support specialists were selected and hired. One staff member commented that one of the best ways of assuring that client need is assessed accurately is in the selection of the support specialists. In State C, the support specialists were from "the area they were going to serve, people who are familiar with the problems in the area." The goal was to select women with life experiences similar to women from the targeted culture. To help facilitate this awareness, time during the training was spent discussing the needs of women in the different communities.

The second technique for assessing need is through the results of the assessment instruments. In the training, the assessment instruments were reviewed and the purpose of each in determining client need was discussed. The manner in which this was carried out was a discussion of the logic model. The logic model lists the areas (referred to as domains) that have been shown to be important in helping women decrease, stop and/or stay off of drinking alcohol during a pregnancy. The domains are linked to assessment instruments and finally activities for the support specialists to engage in to help women who are struggling in those specific domains. For example, social support is a domain. There is a measure of social support and activities to help increase a client's social support if her score on the measure shows that she is in need of support. The logic model was briefly reviewed and each support specialist received a copy of the model. The model highlighted areas of client need and provided "a matrix for identifying/assessing areas of need."

In addition, motivational interviewing training was used to assist in determining client need. It was thought that motivational interviewing would provide the support specialists with "some tools with which to assess need, as well as to begin the action process of getting the participants to do something about their needs." There was also time in the training devoted to brainstorming resources in different geographic areas so that the support specialists would have a list of resources for meeting client need.

Additional training sessions were held throughout the year. At these trainings, further needs assessment information was discussed. One example given was that some support specialists had a difficult time matching client needs with the common curriculum activities mentioned above, so further training emphasized this area.

#### State C support specialists

The support specialists stated that needs assessment in the training focused on the assessment instruments and the use of the healthy prevention booklet. This booklet was given in addition to the 4-State FAS curriculum materials. They also received training on FAS and working with substance abuse. These topics were most important in need determination for one of the support specialists. She also mentioned the training for motivational interviewing as assisting in need determination. In the training, they discussed that the support specialists would receive written results of the assessment instruments along with needs information based on those results from the state staff. Additional educational materials assisted with needs assessment identification. These materials included pamphlets about substance abuse and other pertinent issues and the video titled "Sacred trust: Protecting your baby from FAS". One of the support specialists remembered interaction around the test instruments, but no role plays or group activities. Both of the support specialists considered the training helpful. The information about assessment and FAS were considered especially pertinent.

### Determining need in the field

#### State C staff

In a similar fashion to State A, support specialists in State C were with the clients when they completed the assessment instruments. Also, the specialists completed the ASI interview and sent completed assessments to the state staff. Staff stated that "by the time they have gone through the ASI with each participant, the support specialists have a pretty good idea of the person's needs, at least as far as the ASI determines." The computer returned a report sheet on the assessments and the ASI had a separate report sheet. The state staff added comments to the support sheets and mailed them back to the support specialists. The support specialists were free to call state staff with any questions they had about the instruments, the need that was brought out in the report sheets, or with any other questions. State staff reported that the support specialists routinely called them with questions.

In addition, site supervisors who assisted the support specialists were seen as helpers in need determination in the field. The state staff thought that support specialists turned to their supervisors often to assist in assessing need. State staff reported that the participants also often spontaneously tell the support specialists the needs that they have.

#### State C support specialists

For the two specialists in State C, need determination has been constructed in the field in different ways. Both support specialists mentioned that after the assessment instruments are completed they are sent back to state staff who then send information on client need back to the specialists. They both also felt that ongoing interaction between themselves and other staff members offer continuing support and help in the needs assessment process.

For one of the support specialists, the results from the assessment instruments provide the sole method for need determination. The receipt of information from the state was seen as the beginning of the program with her clients. She stated that she began the discussion by saying "this came back on the assessment, can I offer you support on this?"

An example she relayed was a client who had high scores on the instruments that assessed depression. She discussed this finding with the client who stated that she was depressed due to financial difficulties. The support specialist helped the participant to develop a budget and the participant relayed on her next visit that her financial situation was improving. The specialist noticed that the depression also seemed to be decreasing.

Regarding how need is done over time, the specialist said that "each visit involves going over activities and areas of need highlighted in their personal plan. This allows for a structured and ongoing assessment of needs." The support specialist felt that this process worked well.

The other support specialist stated that her experience with other agencies and lengthy work history in the community were the keys to helping her understand and assess needs. She believed that this work experience and her familiarity with the community was why she was hired. This specialist also used needs assessment tools from her previous job sites to help her.

She also mentioned that working with and receiving community referrals also added dimension to the needs assessment process. A referring agency might have provided her with information about the needs of a client when they made a referral. Also, past work experience with the needs common to an at-risk population was useful in knowing where to begin in assessing needs. Over time, she felt that the needs assessment must be flexible and ongoing as women find support resources and their immediate needs are met or new needs arise. She stated that at first "it may be electricity, then gas vouchers, then food money, and finally parenting tools." Both support specialists felt that the process that they used to determine need worked well.

## Discussion

There are many programmatic similarities across the three consortium states. For example, all three states used support specialists, all three states gathered a core set of data on client determinants and support specialist activities, and all three states used common intervention activities. Although the three states differed on their exact definition of a support specialist, all states saw these staff members as women who were familiar with their communities and who had an ability to work closely with high-risk pregnant women. One of the consortium activities that was developed separately by each state was how client need for the intervention group would be determined. To understand how support specialists were trained to determine need and how need was determined in the field, semi-structured interviews were conducted with state staff and support specialists in three states.

In State A, state staff and support specialists stated that client needs were identified primarily through the assessment instruments. The developers of the ASI stated that "upon completion there is generally the sense that the interviewer has a realistic appraisal of the patient's status in each of these areas"[21] (p. 421). These areas are medical condition, employment, drug use, alcohol use, illegal activity, family relations, and psychiatric condition. In State A, a great amount of time and care were taken to develop support plans specific to needs that arose from the assessment instruments. These support plans were sent back to the support specialists quickly and state staff took time to follow up on these plans with the support specialists. The support specialists believed that this system was effective and useful for them. In addition, state staff felt that motivational interviewing and case management information from the training assisted the support specialists with determining need. One support specialist mentioned motivational interviewing as a helpful adjunct for determining need.

In State B, state staff mentioned that support specialists completed the Difference Game, a card-sort game developed to identify client need from the viewpoint of the client. In addition, motivational interviewing assisted support specialists in their work. The Difference Game was completed early in the process and the needs that arose from the game were reevaluated over time. All support specialists emphasized that they assessed need from the viewpoint of their client, though one specialist did not mention the difference game. They all stated that they received support from other staff members and supervisors and that this was an effective method for determining need.

In State C, state staff stated three things that help in need determination. First was hiring support specialists who were familiar with their community. Second was the information from the assessment instruments. Third were educational materials. Support specialists who were intimately familiar with the community they work in, who were aware of women's needs and of resources to meet those needs were seen as the most important mechanism for accurate needs assessments. Although all three states worked to hire women from the community who would be able to relate to the clients, only state staff from State C mentioned this as a part of the needs assessment. State staff sent reports of assessment instruments with comments on areas of need back to support specialists. The two support specialists described conducting need determination in the field in different ways. One support specialist relied on information provided by the assessment instruments. The other specialist relied on past experience, knowledge of the community, need determination tools from past work, and information from referral sources to determine need. Both specialists stated that their methods seemed to work well for them.

The projects in States A and C had the support specialists collect data for the assessment instruments. These two states relied on their state staff to provide need information to their support specialists based on results from the assessment instruments. It was also understood that support specialists gathered an understanding of client need by conducting the ASI interview. State A's system for feeding back need data was much more structured than State C's.

Unlike States A and C, data from assessment instruments was not collected by support specialists in State B. In State B, the main use of the assessment instruments was to track change in clients over time. Completed assessment instruments were sent to the evaluation team which was off-site from the state staff. Some support specialists in State B received information from the ASI and other assessments from their supervisors, though this was not uniform across the four specialists or uniform over time within the specialists.

In State A, the need identification and support plan were directly linked with the common activities that were developed for the consortium. The common activities were in turn directly linked with the logic model and assessment instruments. For example, there was an assessment to measure social support and common activities aimed at increasing social support.

In State B, some of the Difference Game cards mirrored the information obtained in the assessment instruments. For example, three of that cards state that "it would make a difference if I had" drug or alcohol treatment, someone to talk to about the things that worry me, a real friend. These cards would be linked back to the ASI and the social support assessment. Many of the items in the difference game focus on primary needs – for example, food, housing, sleep, clothes, safety. The underlying assumption for the State B group was that by letting women choose their own needs they would see that the support specialists cared about them and this would enable them to be open to discussing their alcohol and/or drug use. The other assumption was that only when basic needs such as food and shelter are met can higher needs be attended to [3].

In State C, there was a combination of need coming from the client and need coming from the assessment instruments. State staff felt that they hired women who were knowledgeable about needs in their community and this was combined with information from the assessment instruments. The two support specialists in this state used different methods for determining client need.

These data are limited by a number of factors. First, information on training was gathered months after the training occurred and answers may have been affected by recall bias. However, this method was necessary in order to gather insight into the differences between need assessment information received in training and how needs were assessed with clients in the field. Second, in general, it is better to gather survey data via in-person interviews versus telephone interviews. Data is thought to be of higher quality and interviews can be longer with in-person interviews. Fortunately, the interviewers had easy access to the respondents and could conduct follow-up interviews if new questions arose while filling in notes after the interviews or if questions came up in the data analysis phase. Lastly, qualitative data analysis is always impacted by the experiences and viewpoints of the people conducting the data analysis. To lessen this impact and increase validity, respondents read the portions of this manuscript that related to the information they provided and offered suggestions and changes.

## Conclusion

This paper provides process evaluation information of the needs assessment method. Process evaluations provide quality assurance, present opportunities to enhance interventions by providing information on areas to modify or adjust, and make available practical and useful answers to questions that cannot be answered by outcome evaluation alone[7,22-24]. Although there is a plethora of published information on intervention outcomes, there currently exists a "dearth of conceptual underpinnings and methodologies for conducting process evaluations"[25]. Also, no published studies were located that assessed the difference between needs assessment training and application in the field. This article should be useful to those designing, implementing, and evaluating health programs, specifically those working in health programs that conduct client needs assessments.

## Competing interests

The author(s) declare that they have no competing interests.

## Authors' contributions

SC&LS conducted the interviews. All authors contributed to the study design, data analysis and drafting of the manuscript. All authors read and approved the final manuscript.
